# Effects of Erosion Forms and Admixture on Cement Deterioration Characteristics in Sulfate Environment

**DOI:** 10.3390/ma19050885

**Published:** 2026-02-27

**Authors:** Ying Chen, Peng Liu, Zhiwu Yu

**Affiliations:** 1 School of Civil Engineering, Central South University of Forestry and Technology, 498 Shaoshan Road, Changsha 410004, China; chenying8307@163.com; 2 School of Civil Engineering, Central South University, 22 Shaoshan Road, Changsha 410075, China; zhwyu0512@163.com; 3 National Engineering Research Center of High-Speed Railway Construction Technology, 22 Shaoshan Road, Changsha 410075, China

**Keywords:** admixtures, microstructure, compressive strength, chemical attack, durability

## Abstract

The effects of solution concentration, admixtures, erosion form, and age on the flexural and compressive strength of Portland cement subjected to a sulfate environment were investigated, and the corresponding relationship between the mechanical properties and influencing factors of the cement specimen was proposed. Furthermore, the phase components, crystal morphology, microstructure, and morphology of erosion products were also investigated. The research findings indicate that the flexural and compressive strengths of specimens subjected to a sulfate environment for 4 months decreased as the content of admixture increased. There exists a GaussMod function between the content of fly ash and flexural strength of a specimen subjected to a sulfate environment, and the Boltzmann function can be used to characterize the variation between the slag content and compressive strength of the specimen. After being attacked by a saturated sulfate solution, the strength of specimens with fly ash increased at first and then decreased as the content of fly ash increased. In the semi-immersion erosion form, the strength of the specimens containing admixture that were attacked by sulfate was lower than that of the control sample. Admixture can observably change the morphology and microstructure of the specimens. Rodlike and slab-like sulfate erosion products can be easily observed in the specimens containing admixture that were attacked in the semi-immersion form. This is significant for further research on the mechanism and evolution process of concrete sulfate erosion and for predicting durability and conducting an operational life assessment of concrete constructions subjected to a sulfate environment.

## 1. Introduction

Although cement and concrete are the most widely used building materials in civil engineering construction, their durability and service life are significantly affected by the external environment. Among the influencing factors of durability, sulfate attacks are one of the most severe and rapid mechanisms that cause concrete structures to degrade [1,2]. Sulfate attacks can induce the deterioration of the microstructure, composition, and morphology of hydration products, which accordingly shortens the service life and reduces the durability of concrete structures [3]. Therefore, sulfate attacks have a significant effect on the reliability of a structure [4,5,6]. While research efforts have mainly been focused on sulfate attacks on concrete since 1920, the mechanisms and deterioration of sulfate attacks have not been expounded convincingly [7]. The widespread occurrence of sulfate erosion and the damage caused by it have prompted researchers to conduct studies on its deterioration mechanism and prevention over the years [8,9]. Therefore, research on cement and concrete materials attacked by sulfate is of great significance.

Sulfate attacks are a process that results in destructive damage to concrete due to the formation of expansive reaction products and salt crystallization within concrete exposed to an external sulfate environment [10,11]. There are mainly two forms of sulfate attacks: physical and chemical sulfate attacks [12]. Chemical sulfate attacks involve a chemical interaction between sulfate salts and cementitious minerals, and physical sulfate erosion usually refers to salt scaling, salt hydration, or salt weathering [13]. Many studies on cement and concrete that are attacked by sulfate have been carried out [14] which mainly focus on cementitious materials, admixture, the concentration and kind of sulfate solution, the water-to-cement ratio, load, pH value, environmental factors, the erosion mechanism, and the deterioration model [15,16,17,18,19,20,21,22,23,24]. For example, Haynes proposed that a concrete sulfate attack consisted of the coupled effects of physical and chemical forces, which could be verified by the change in alkaloid content, as well as ettringite, gypsum, and calcium magnesium silicate hydrate. Hill [25] pointed out that resistance to sulfate erosion could be enhanced when concrete was composed of 65% slag. Biczok [15] proposed that the main product of concrete subject to sulfate attack was ettringite when the sulfate ion concentration was lower than 1 g/L, and it was gypsum when it was higher than 8 g/L. Santhanam [26] discussed the effects of the concentration and temperature of sodium and magnesium sulfate solutions on the expansion of cement mortars, and the results indicated that the concentration effect for both solutions was modeled as a rate law, while a modified Arrhenius relationship was used to account for the temperature of the sodium sulfate solution. Erlin and Stark [27] first reported engineering failure in the western United States when concrete was attacked by thaumasite (CaSiO_3_·CaCO_3_·CaSO_4_·15H_2_O), and some researchers [28,29,30] defined the thaumasite form of sulfate attack on concrete. Moreover, models of diffusion–reaction, expansion degradation, and STADIUM for concrete sulfate attacks were proposed [31,32,33].

Although large amounts of research on cement and concrete sulfate attacks have been performed, the mechanism of sulfate erosion, the deteriorating law, and the characteristics of the erosion products of cement-based materials attacked by sulfate have not been thoroughly investigated. Sulfate attacks of concrete can be observably affected by material composition, environmental factors, and erosion form [34]. Existing studies almost only discuss a single influencing factor of sulfate attacks, which makes it difficult to conduct qualitative and semi-quantitative analyses of concrete sulfate attacks. Therefore, it is difficult to compare the achievements obtained by researchers with each other. Moreover, the changing relationship before and after sulfate attacks between the macro-performance and microstructure of concrete has been little investigated. The purpose of this research is to discuss the effects of sulfate solution concentration, admixture, erosion form, and age on the compressive and flexural strengths of Portland cement subjected to a sulfate environment, as well as the internal relationship and variation rules of the strength of the specimens. Also, the morphology, phase components, crystal form, and microstructure of hydration products were studied using X-ray diffraction (XRD), Fourier transform infrared spectroscopy (FTIR), and an Environment scanning electron microscope (ESEM), in detail. The achievements obtained can provide support for the engineering application of cement and durability assessment of concrete structures subjected to a sulfate environment.

## 2. Experimental Materials and Methods

### 2.1. Experimental Materials

**Figure 1:**
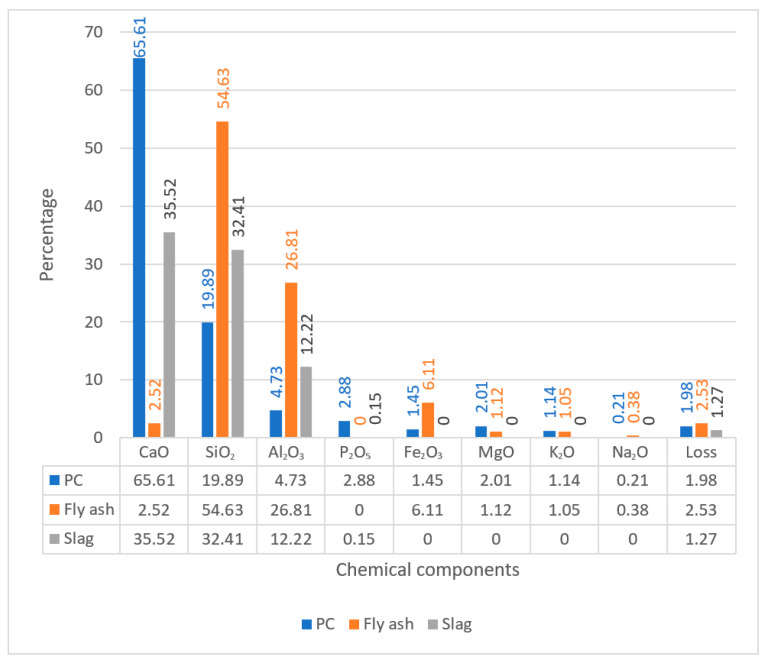
Chemical components of cements/%.

The main raw materials are as follows. Portland cement (PC) of P•O 32.5 was produced by Pingtang Cement Co., Ltd. (Changsha, China) I class fly ash was purchased from the Hunan Xiangtan power plant (Xiangtan, China), and S95 slag was obtained from Hunan Lianyuan Iron and Steel Group Co., Ltd. (Loudi, China). Industrial anhydrous sodium sulfate with a purity of 99% was purchased from a local market. The chemical compositions and physical properties of cementitious materials are listed in Figure 1 and Table 1, respectively.

### 2.2. Specimen Preparation, Experimental Process, and Test Devices

According to GB175-2007 Common Portland Cement [35] and GB/T 1346-2011 [36] Test Methods for Water Requirement of Normal Consistency, Setting Time and Soundness of the Portland Cement [36], paste specimens were cast with the dimensions of 40 mm × 40 mm × 160 mm and a water cement ratio of 0.28. All specimens were demolded after 24 h and cured for 28 d at a temperature of 20 ± 1 °C and a relative humidity of 95 ± 5%. Without special instructions, the control sample was defined as the paste specimen that was cured in standard conditions (i.e., temperature of 20 °C and a relative humidity of 95%) for the same curing time without admixture or sulfate

**Figure 2:**
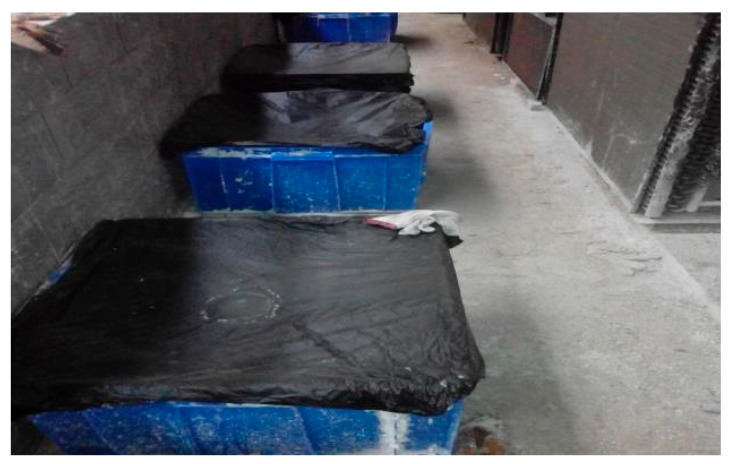
Concrete immersion test in sulfate solution.

Solutions of varying concentrations (i.e., 1%, 5%, 10%, and saturated solution) were prepared. In order to obtain a saturated solution, 30 wt% sodium sulfate was dissolved in liquid water. After 28 d of curing, the specimens were put into a solution chamber at a temperature of 25 °C. In the experiments, the specimens were completely immersed in sulfate solution. Firstly, solutions of various concentrations (i.e., 1%, 5%, 10%, and saturated solution) of sulfate were prepared and poured into different test chambers separately. The specimens were immersed in the solution mentioned above. The specimens were positioned more than 2 cm apart in order to make the sulfate solution uniform. Then, the test chambers were covered with plastic films in order to avoid water vaporization, as shown in Figure 2. Finally, the specimens were taken out from the test chambers and placed in a laboratory of environment simulation, in which the specimens were dried naturally at a constant temperature of about 20 degrees centigrade and 50% RH for 3 days. The strength of specimens with different erosion ages (i.e., 2, 4 months) was tested. Furthermore, the sulfate solution was changed weekly to ensure that the concentration of the solution remained constant. Another group of sulfate erosion experiments with semi-immersion was carried out for comparison, and the procedure was the same as that used for complete immersion. The use of the semi-immersion form means that the lower half of concrete specimens was vertically immersed in the sulfate solution above and the upper half was exposed to air. Compared with the semi-immersion form, the partial immersion form of specimens with an immersion depth of 20 mm was used to investigate salt crystallization. An introduction to the specimens and notations can be found in Appendix A, Table A1.

The main test equipment is as follows. Quanta-200 Environment Scanning Electronic Microscope (ESEM-EDS, FEI, Hillsboro, OR, USA) with an amplification of 600,000 times was used to measure the microstructure. A BD-86 X-ray diffractometer (XRD, BRUKER AXS GMBH, Karlsruhe, Germany) with an angular range of 5~120° and a minimum scanning step size of 0.01° was employed to investigate the crystal form. A WQF-510A Fourier Transform Infrared Spectrometer (FTIR, Beijing Beifen-Ruili Analytical Instrument (Group) Co., Ltd., Beijing, China) with a wave number range of 350~7800 cm^−1^ and distinguishability of 1 cm^−1^ was used to determine the functional group of hydrations. Moreover, a YAW-300D (Jinan Hensgrand Instrument Co., Ltd., Jinan, China) was used to measure the flexural and compressive strengths of specimens. Before microstructural analysis, the samples were prepared for different areas of the tested specimens and immersed in absolute alcohol to terminate the reactions. During the ESEM (SEM)-EDS analysis, the specimens were removed and vacuum-dried. Subsequently, an environmental scanning electron microscope (ESEM) was employed to conduct an analysis of the micro-morphology and structure, and an EDS energy spectrum analysis of the elements was also carried out.

In order to obtain reliable data and draw statistically significant conclusions, three samples at least are used for each test series. The standard deviation or error of the results for mechanical properties is less than 5%.

## 3. Results and Discussions

### 3.1. Effects of Admixtures on Strength of Specimen Attacked by Sulfate

**Figure 3:**
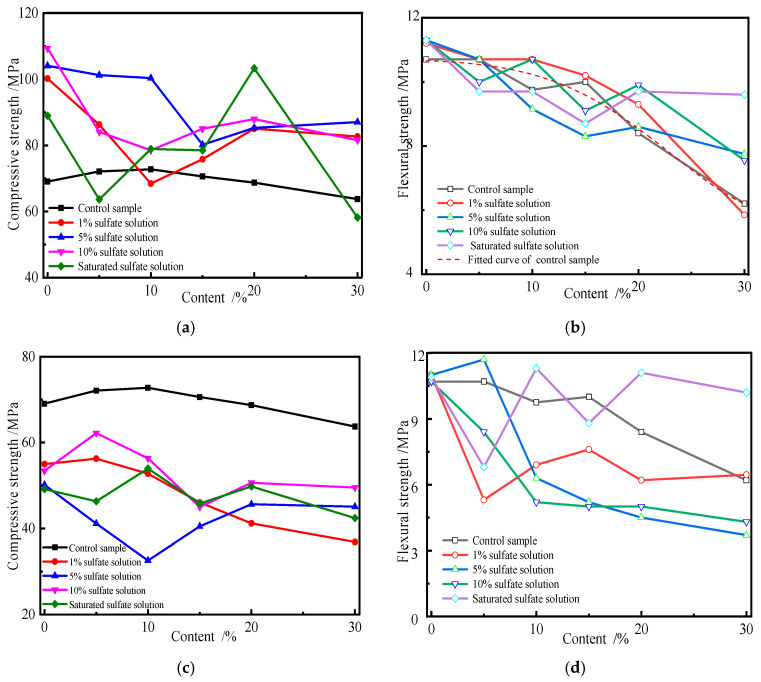
Variations in flexural and compressive strengths of specimens attacked by sulfate with fly ash. (**a**) Compressive strength for 2 months. (**b**) Flexural strength for 2 months. (**c**) Compressive strength for 4 months. (**d**) Flexural strength for 4 months.

Previous studies have shown that the addition of admixture (i.e., fly ash or slag) micropowder not only reduces the amount of cement used but also inhibits the formation of calcium aluminosilicates through the “volcanic ash effect”, thereby enhancing concrete’s resistance to sulfate. The new anti-sulfate admixture, by introducing limestone micropowder and fly ash, performs exceptionally well in resisting sodium sulfate erosion, showing only slight expansion, and significantly improves the concrete’s corrosion resistance. In order to discuss the influence of the kind and content of admixtures on the strength of cement specimens attacked by sulfate, the flexural and compressive strengths of specimens subjected to a sulfate environment for different erosion ages were measured. Figure 3 shows the change curves of the flexural and compressive strengths of the specimens with an increase in the fly ash content.

In Figure 3, the changes in the flexural and compressive strengths of specimens attacked by sulfate for 2 months are shown. The flexural strength of the control sample slightly increases at the initial stage of sulfate attack, but the corresponding compressive strength significantly increases. The reason for this may be the following. The calcium hydroxide (CH) generated by the hydration of Portland cement is directionally arranged and enriched together in the interfacial transition zone, which has a negative effect on the microstructure and mechanical properties of hardened cement paste. The intruding sulfate ion can be reacted with CH and used in the generation of expansive products in cement, such as ettringite (AFt) and gypsum, which can fill the pores, thereby decreasing the porosity and improving the microstructure of the system. Moreover, AFt also has a higher mechanical strength than other hydration products. Thus, sulfate erosion on cement plays a positive role in the performance of hardened cement paste when expansive products are properly generated. This is the reason why the strength of cement specimens attacked by sulfate for 2 months increases significantly, as seen in Figure 3a. The flexural strength of cement specimens charged with fly ash decreases with an increase in fly ash content, but the corresponding compressive strength increases first and then decreases. The compressive strength of the specimens charged with fly ash reaches the maximum value when fly ash substitutes for 10% cement. When the concentration of sulfate solution is below 1%, no significant variation is observed from the flexural strength of the control sample, but the corresponding compressive strength observably increases. Once fly ash is used to substitute cement, the flexural and compressive strengths of specimens increase, and they are larger than those of the control samples. Fly ash consists of active components including calcareous, aluminous, and silicious materials, which can be activated by CH and produce hydration products [37]. Simultaneously, the hydration products of cement can react with sulfate ions attacking from the outside and generate expansive substances, i.e., AFt and gypsum. They can fill in the pores of specimens, enhance compactness, and improve the composition and microstructure. The outcome of sulfate attacks is represented as variations in the flexural and compressive strengths of the specimens. So, sulfate erosion plays a positive role in the properties of specimens in a low-concentration sulfate solution and for a short erosion duration [38]. The flexural strength of the specimens decreases with an increase in fly ash content when the concentration of sulfate solution is 5%. The flexural strength of specimens is larger than that of the control sample when the substitute amount of fly ash is less than 10%. Conversely, the change in the compressive strength of the specimens is not significant. The compressive strength of the specimens decreases with an increase in the substitute amount of fly ash, but its value is larger than that of the control sample. When the concentration of sulfate solution is 10% or saturated, the compressive strength of specimens containing fly ash is larger than that of the control sample, but its corresponding flexural strength decreases slightly. In summary, the compressive and flexural strengths of specimens charged with fly ash increase when the specimens are attacked by sulfate for 2 months. Conversely, the corresponding strengths of specimens attacked by sulfate for 4 months decrease with an increasing substitute amount of fly ash, and they are less than that of the control sample. Moreover, the compressive strength of the specimens decreases with an increasing concentration of sulfate solution. By fitting the measured data in Figure 3, the flexural strength of the specimens can be characterized by a GaussMod function, written as Equation (1).

(1) $$y=y_{0}+E/t_{0}\cdot exp\left(0.5\cdot {\left(w/t_{0}\right)}^{2}-\left(x-x_{c}\right)/t_{0}\right)\cdot \left(erf\left(W\cdot z\right)+1\right)$$

(2) $$z=\left(x-x_{c}\right)/w-w/t_{0}$$

where *x* is the admixture substitute amount for cement, and *y* is the flexural strength of the sulfate erosion specimens. *W*, *E*, *t*_0_, *y*_0_, *w*, and *x_c_* are fitted parameters.

Figure 3 also shows that the strength of the specimen decreases with an increasing erosion age, and the corresponding strength of the specimen containing fly ash is less than that of the control sample. This is mainly because of the double effects of sulfate attack on the specimen. On the one hand, the sulfate ions are expended and react with hydration products including CH, calcium silicate hydrate (CSH), and calcium aluminate hydrate (CAH). On the other hand, some expansive substances, such as ettringite and gypsum, can be generated by sulfate attacks. If the coupling effects of sulfate corrosion can fill cracks, reduce porosity, and improve compactness and the microstructure, then sulfate erosion can play a positive role in the specimen’s performance. Conversely, sulfate has a negative effect on the performance of the specimen when micro-damage and cracks are caused by expansive substances. With increasing erosion time, more sulfate ions intrude into the specimen. Therefore, more hydration products generated by cementitious minerals can be expended, which reduces the pH value and disturbs the chemical thermodynamic equilibrium state of the system. Moreover, more and more expansive substances generated with sulfate attacks can induce micro-damage and cracks, which are caused by the enormous pressure of crystallization [39]. The greater the substitution of fly ash, the smaller the amount of CH. Therefore, the chemical thermodynamic equilibrium state of the system can be disturbed easily. Figure 3c,d also show that the influence rules of the strength of the specimen with various substitute amounts of cement are similar, but there are differences in the variation and percentage. Macroscopically, variations in the mechanical properties of specimens are caused by the variation in the composition, microstructure, and morphology of specimens before and after sulfate erosion.

**Figure 4:**
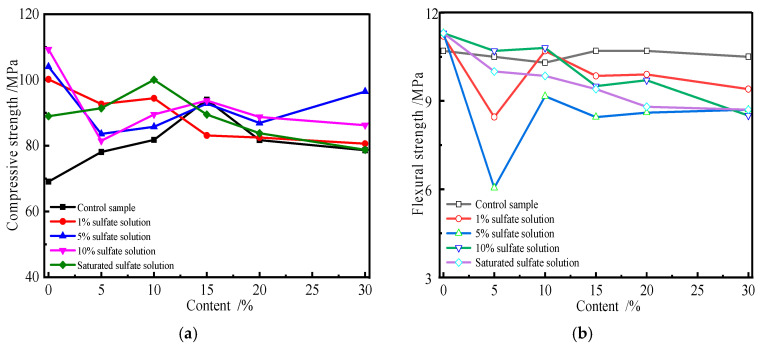

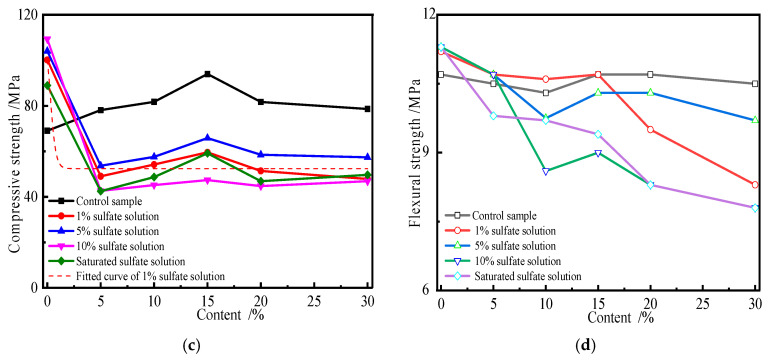
Variations in flexural and compressive strengths of specimen subjected to sulfate attack with slag. (**a**) Compressive strength for 2 months. (**b**) Flexural strength for 2 months. (**c**) Compressive strength for 4 months. (**d**) Flexural strength for 4 months.

Moreover, the influence of erosion age, the concentration of sulfate solution, and admixture on the strength of specimens was also studied. The corresponding results are shown in Figure 4.

As seen from Figure 4a,b, the compressive and flexural strengths of the control sample first increase and then decrease. With an increasing sulfate solution concentration and substitute amount of slag, the flexural strength of the 2-month sulfate erosion specimen decreases, and the strength value is lower than the control sample’s. Simultaneously, the change trend in the compressive strength of the specimen is similar, but the corresponding strength value is larger compared to that of the control sample. The flexural strength of the specimen eroded by 1% sulfate solution decreases, and the variation is more significant when the substitute amount of slag is 5%. This is attributed to both the positive and negative effects of sulfate erosion on the performance of the specimen. The difference in mechanical characteristics between compressive and flexural specimens is the dominant influencing factor leading to a difference in trends and the final specimen; that is, the flexural and compressive strengths of the specimen are affected by tensile and compression failure, respectively. Figure 4c,d show that the flexural and compressive strengths of the specimen containing slag decrease after sulfate erosion for 4 months, and they are lower than those of the control sample. Compared with compressive strength, the flexural strength of the sulfate erosion specimen is more significant. With increasing erosion time, more sulfate ions intrude into the specimen. Therefore, sulfate erosion negatively affects the performance of the specimen. Figure 4 also shows that there exists a linear or exponential function between the flexural strength of the specimen and the content of admixture, represented as Equation (1). However, a Boltzmann function can be used to characterize the changes between the compressive strength of the specimen subjected to sulfate attack for 4 months and the substitute amount of slag, written as Equation (3).

(3) y=A+B−A(1+exp(x−x0D))

where *x* is the slag substitute amount for cement, and *y* represents the flexural and compressive strengths of sulfate erosion specimens containing slag. *A*, *B*, *D*, and *x*_0_ are fitted parameters.

By comparing Figure 3 and Figure 4, it can be concluded that the strength change in the specimen with slag is more significant than that in fly ash under the same erosion conditions. This is because the active components of slag are more easily activated by CH and generated hydration products. The sulfate ion intruding into the specimen can expend CH and reduce the pH value of the system, which may destroy the chemical thermodynamic equilibrium state. Therefore, resistance to sulfate attacks decreases. Sulfate erosion has a positive effect on the performance of the specimen in a low-concentration sulfate solution and with a short erosion time. Conversely, it has a negative effect.

### 3.2. Effect of Concentration of Sulfate Solution on Strength of Specimen

**Figure 5:**
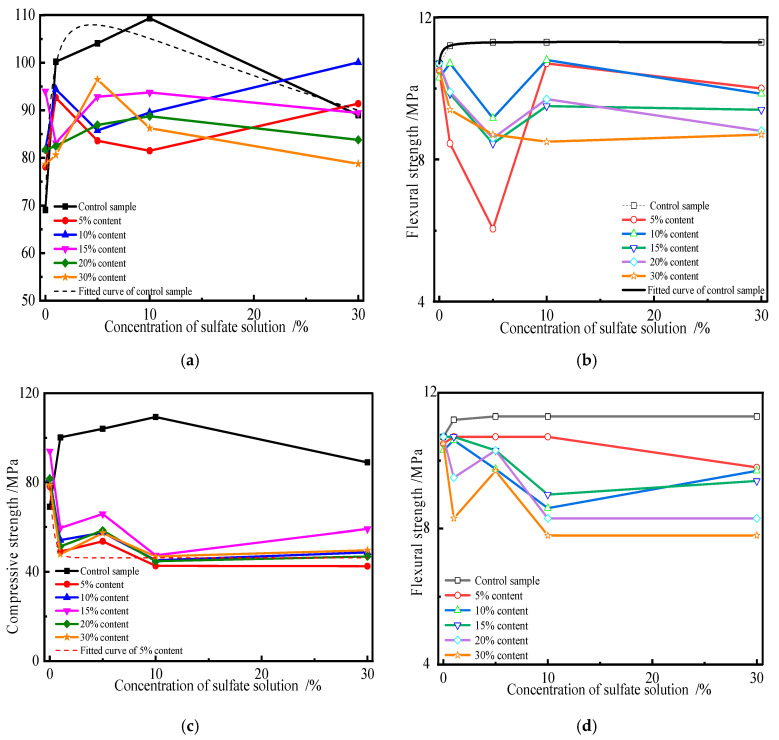
Variation in compressive and flexural strengths of specimen with concentration of sulfate solution. (**a**) Compressive strength of specimens with fly ash. (**b**) Flexural strength of specimens with fly ash. (**c**) Compressive strength of specimens with slag. (**d**) Flexural strength of specimens with slag.

The effect of the concentration of sulfate solution on the strength of the specimen in the complete immersion form was studied, and the changes in the compressive and flexural strengths of the sulfate erosion specimen after 4 months are shown in Figure 5.

Figure 5a indicates that the compressive strength of the control sample increases at first and then decreases with an increasing concentration of sulfate solution, and the value of the compressive strength of the specimen subjected to 10% sulfate solution for 4 months reaches the maximum. However, the flexural strength of the specimen gradually increases and tends to be constant as the concentration of sulfate solution increases, as shown in Figure 5b. Simultaneously, the compressive strength of the sulfate erosion specimen increases at first and then decreases with an increasing substitute amount of fly ash. Figure 5c,d indicate that the compressive and flexural strengths of the sulfate erosion specimen after 4 months increase and tend to be constant with an increasing concentration of sulfate solution. There is a Nelder functional relationship between the concentration of sulfate solution and the strength of the specimen containing fly ash, written as Equation (4). However, the Boltzmann function can be used to characterize the strength variation in the specimen mixed with slag, represented as Equation (3).

(4) y=x+ab+cx+a+dx+a2

where *x* is the fly ash substitute amount for cement, and *y* is the flexural strength of sulfate erosion specimens mixed with fly ash. *a*, *b*, *c*, and *d* are fitted parameters.

### 3.3. Effects of Erosion Form on Strength of Specimen

**Figure 6:**
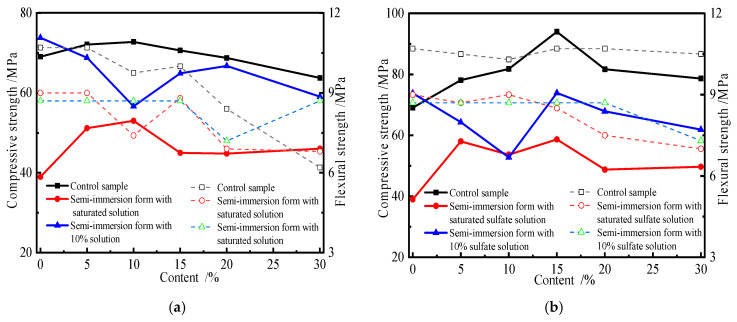
Variation in strength of specimens with slag and fly ash attacked in semi-immersion form. (**a**) Fly ash. (**b**) Slag.

The effects of erosion forms, i.e., complete and semi-immersion forms, on the mechanical properties of the specimen affected by sulfate erosion were studied. Figure 6 shows the change in the compressive and flexural strengths of the specimen subjected to sulfate attacks in the semi-immersion form. The specimens containing fly ash and slag were immersed in 10% or saturated sulfate solution for 4 months.

Figure 6a shows that the strength of specimens subjected to 10% sulfate solution in the semi-immersion form decreases as the substitute amount of fly ash increases. However, the strength variation trend in the specimens attacked by saturated sulfate solution increases first and then decreases. Compared with 10% sulfate solution, the saturated sulfate solution has a noticeable influence on the strength of the specimen. As Figure 6b shows, the compressive strength of the specimen attacked in the semi-immersion form first increases and then decreases with an increase in the substitute amount of slag. But the corresponding flexural strength of the specimen decreases with an increase in the substitute amount of slag. In the semi-immersion form, the flexural and compressive strengths of the specimen containing admixture are all lower than those of the control sample. The change in the specimen containing slag is more significant than that in fly ash. Although the trends in the compressive and flexural strengths of the specimen attacked by various concentrations of sulfate solution are similar, the change and percentage are different. There is a difference in the compressive strengths of the specimens affected by sulfate erosion between the 10% and saturated solutions. The higher the concentration of sulfate solution, the greater the concentration gradient. More and more sulfate ions can intrude into the specimen, so the mechanical variation in the specimen is more significant. Comparing Figure 3 and Figure 4, it can be included that the decrease in the flexural strength of the specimen containing admixture attacked in the semi-immersion form is less than that for the specimen attacked in the complete immersion form. This is because the half of the specimen immersed in sulfate solution is attacked by sulfate, so salt crystallization occurs when the specimen’s surface is above the liquid level. Therefore, deterioration focuses on the salt crystallization zone. Figure 6 also indicates that the relationship between the specimen’s flexural or compressive strength and admixture substitute amount can be characterized by Equations (1) and (4), respectively.

To reveal the deterioration rule of the sulfate erosion specimen in the semi-immersion form, the characteristics of salt crystallization on the surface of the specimen immersed in 10% and saturated sulfate solutions were investigated. The immersion depth along the length of the specimen was set as 20 mm. The container had an opening at the top which could be used for ventilation, and the sulfate solution was replaced weekly.

The results show that there are plenty of salt crystals on the surface of the specimen attacked by saturated sulfate solution in the partial immersion form. The length of the salt crystallization area on the surface of the specimen ranges from the liquid level to 3/5 of the specimen length, when the specimen is eroded by 10% sulfate solution. The amount and range of salt crystallization are smaller than those of the specimen attacked by the saturated solution. This because only a part of the specimen is immersed in solution, and the rest of the specimen is exposed to an external environment. The sulfate solution transfers along the surface of the specimen under capillary action and skin layer action, so the solution in the pores of the specimen surface tends to be saturated under the action of evaporation. As a result, plenty of salt crystallization takes place on the surface of the specimen [40,41]. A difference in the concentration of solutions induces a difference in the amount and range of salt crystallization on the specimen surface. The morphology of salt crystallization differs on the surface of specimens containing slag and fly ash. The morphologies of salt crystallization on the surface of the specimen with fly ash are fibrous and flocculent, but they are nubbly and granular for specimens containing slag. This may result from the composition and elements of admixture. The main components of fly ash are siliceous and aluminous materials, but they are calcareous and siliceous materials for slag. The active components of slag and fly ash become activated by CH and are generated into hydration products. The kind and content of ions in the pore solution of the specimen are different, which results in a difference in the activity of ions and the thermodynamic equilibrium parameters of erosion products. Therefore, the kind, morphology, and crystal form of salt crystallization on the surface of the specimen are different. The above results also imply that there exists an interrelationship between materials’ components and the deterioration mechanism of sulfate attacks.

### 3.4. XRD Analysis of Phase Composition of Specimen

(1) Effects of admixture on phase composition of specimen

**Figure 7:**
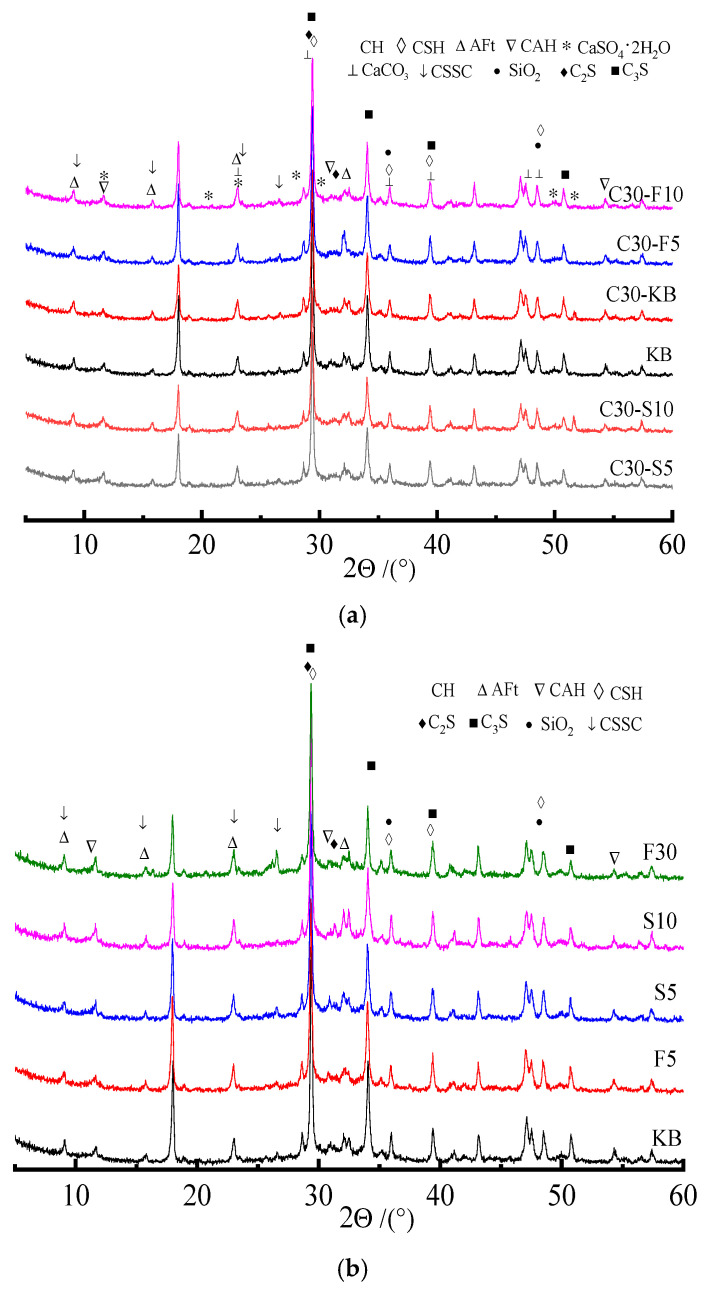
XRD spectra of phase components of specimen before and after sulfate attack. (**a**) Specimens attacked by sulfate. (**b**) Control samples containing admixture not attacked by sulfate.

In order to study the effects of the kind and content of admixture on the erosion products of the specimen, the phase composition of the specimen subjected to saturated sulfate solution for 4 months was investigated. Figure 7 shows the XRD spectra of specimens containing admixture before and after sulfate attacks.

**Figure 8:**
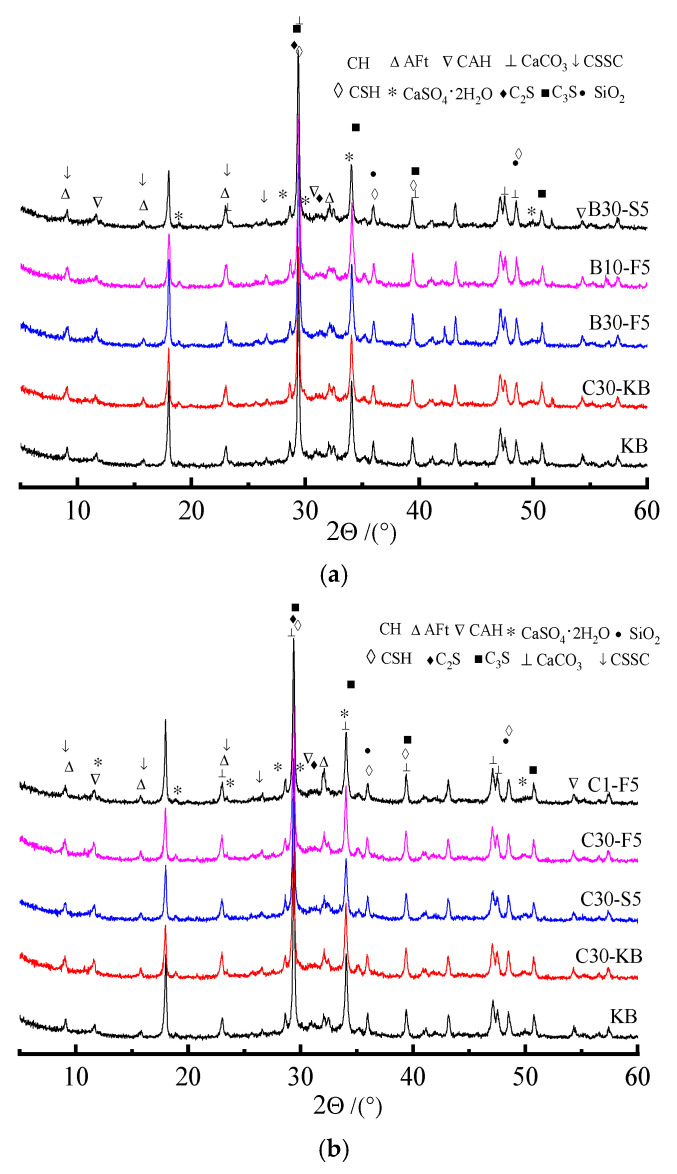
XRD spectra of phase components of specimen subjected to sulfate attack. (**a**) Erosion form. (**b**) Concentration of sulfate solution.

Figure 8a indicates that the variations in the phase composition of specimens before and after sulfate erosion mainly manifest as the appearance or disappearance of diffraction peaks and peak intensity and width. The main hydration products of the control sample (KB) are calcium hydroxide (CH), calcium silicate hydrate (CSH), calcium aluminate hydrate (CAH), and non-hydrated cementitious minerals. However, the erosion products of the specimen subjected to sulfate attacks (C30-KB) are AFt, CAH, CSH, and gypsum. The XRD spectra indicate that the intensity of the diffraction peak of AFt is enhanced, but it is weakened for CH. Moreover, thaumasite (CSSC) and calcite are present. This is perhaps because the sulfate ion intrudes into the specimen, reacts with CAH, and generates a less soluble substance of AFt. The intensity of the diffraction peak of CH for the specimen attacked by sulfate decreases with an increase in the substitute amount of admixture, which implies a decrease in the amount of CH in hardened paste. As shown in Figure 7b, the intensity of the diffraction peak of the CH of the specimen containing slag is lower than that of the specimen containing fly ash, which is related to the difference in the composition and activity of admixture. The difference in diffraction peaks between the control sample (KB) and specimens containing admixture is expressed as the intensity and width of the diffraction peaks of products. The diffraction peak of CH observably decreases, but the variation in the diffraction peaks of CSH and CAH changes slightly. The greater the substitution of admixture, the smaller the amount of CH. The coupling effects of the replacement, eruption, shape, and tiny aggregate of admixture can reduce the directed cleavage of CH, improve the microstructure, and increase the compactness of the specimen. These can have a positive influence on the performance of the specimen with a proper substitute amount of admixture. Compared with fly ash, the activity of slag can be more easily activated by CH, so the corresponding intensity of the diffraction peak of CH decreases remarkably. Moreover, the specimen containing slag has better sulfate attack resistance, which is because slag can be activated by CH to produce more hydration products.

(2) Effects of erosion form and solution concentration on phase composition of specimen

Erosion form and solution concentration have a notable influence on the mechanical properties of the specimen subjected to sulfate attacks. Therefore, the phase components of the specimen subjected to sulfate attacks in the complete and semi-immersion forms for 4 months were investigated, as shown in Figure 8.

Compared to the control sample (KB), the variation in the diffraction peak of erosion products becomes more significant with an increase in the substitute amount of admixture. Comparing the specimens attacked by the semi-immersion form (B30-S5), it can be seen that the intensity of the diffraction peaks of the CH, AFt, and gypsum of specimens (C30-S5) attacked by saturated sulfate solution under complete immersion is weakened and increased, respectively, as shown in Figure 8a. The results mentioned above illustrate that the admixture and erosion immersion form can have a significant influence on phase composition affected by sulfate erosion. By comparing the XRD spectra of the specimens with fly ash attacked in the semi-immersion form (i.e., B30-F5 and B10-F5), it can be seen that the intensity of the diffraction peaks of the CH, AFt, and gypsum of the specimen (B10-F5) attacked by 10% sulfate solution is weakened and enhanced, respectively. Figure 8b indicates that the effects of the concentration of sulfate solution on the phase composition of the specimen containing admixture mainly manifest as the appearance and disappearance of diffraction peaks and peak intensity and width. For example, the intensities of the diffraction peaks of the AFt and gypsum of specimens (C30-F5 and C30-S5) increase, and those of CH, CSH, and CAH decrease. The higher the concentration of sulfate solution, the more notable the intensity of the diffraction peaks of specimens (C30-F5 and C30-S5). This may be because the sulfate ion reacts with hydration products including CH, CSH, and CAH, leading to the generation of AFt and gypsum. Therefore, the intensity of the diffraction peaks of hydration products changes. It can be concluded that the concentration of sulfate solution can affect the composition, kind, and amount of sulfate attack products.

(3) XRD analysis of phase composition of salt crystallization on specimen surface

**Figure 9:**
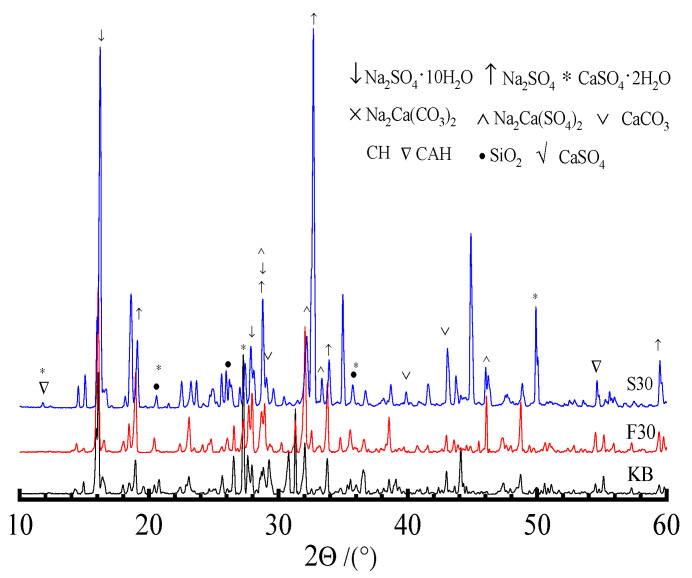
XRD spectra of salt crystallization on specimen surface.

To investigate salt crystallization on specimens’ surfaces, the phase compositions of the specimens subjected to sulfate attacks for 4 months were investigated by XRD, as indicated in Figure 9.

Figure 9 indicates that the differences in the XRD spectra of salt crystallization on the specimen surface are manifested as the intensity and width of the diffraction peaks of erosion products. The main products of salt crystallization on the specimen surface are sodium sulfate, gypsum, sodium sulfate decahydrate, glauberite, sodium gypsum, CH, and CAH. This is because the active components of admixture can be activated and reacted by CH, which results in a change in the kind and content of ions in pore solution. Therefore, the kind and morphology of salt crystallization on the surface of the specimen are different. The main erosion products of salt crystallization on the surface of the specimen containing fly ash are sodium sulfate, gypsum, sodium sulfate decahydrate, and sodium gypsum. Correspondingly, glauberite can be observed from the salt crystallization that occurs on the surface of the specimen containing slag. This may be caused by a difference in the composition and elements of fly ash and slag. Moreover, the characteristic peak of silica can be determined, which may be due to the decomposition of CSH attacked by sulfate. It can be concluded that the erosion products of salt crystallization on the surface of the specimen can be affected by admixture. Some double salts including glauberite and sodium gypsum can be observed from XRD spectra, which changes the traditional concept that the products of salt crystallization are sodium sulfate and sodium sulfate decahydrate.

### 3.5. FTIR Analysis of Phase Composition of Specimen

**Figure 10:**
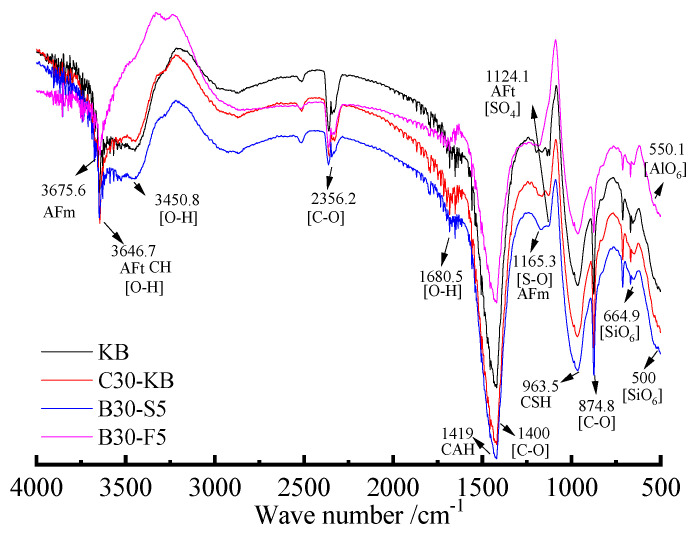
FTIR spectra of phase components of specimens subject to sulfate attack.

FTIR is also employed to investigate phase components and erosion products, as shown in Figure 10.

Figure 10 indicates that many absorption peaks of specimens can be seen; for example, the bending absorption peak at 500 cm^−1^ belongs to the functional group of [SiO_6_]. Those at 669 cm^−1^ and 750 cm^−1^ belong to the telescopic vibration absorption peaks of [SiO_6_]. The weak absorption peak of [AlO_6_] is at 850 cm^−1^, and the bending and stretching vibration absorption peaks of [C=O] are at 875 cm^−1^ and 1400 cm^−1^, respectively. Those at 920 cm^−1^ and 940 cm^−1^ are the absorption peaks of [SiO_4_]. Those at 607 cm^−1^, 1100 cm^−1^, and 1145 cm^−1^ are the stretching vibration absorption peaks of [S-O]. The bending and stretching vibration absorption peaks of [O-H] are at 1680 cm^−1^ and 3200 cm^−1^~3600 cm^−1^, respectively [42]. From what was discussed above, it can be determined that there are very strong and narrow absorption peaks of CH at 3640 cm^−1^. Those at 965~975 cm^−1^ and 980~990 cm^−1^ are the absorption peaks of [SiO_4_] in CSH. Those at 3450 cm^−1^ are the stretching vibration absorption peaks of [O-H], which is a hydration product. The characteristic absorption peaks of CAH range from 1370 cm^−1^ to 1490 cm^−1^, and the corresponding absorption peak assigned to the functional group of [SO_4_] is at about 1120 cm^−1^. Normally, the characteristic absorption peaks of the sulfate group are composed of two parts. Strong absorption peaks range from 1210 cm^−1^ to 1040 cm^−1^, and moderate absorption peaks range from 1100 cm^−1^ to 1250 cm^−1^. As mentioned above, 550 cm^−1^ is assigned to the absorption peak of [AlO_6_] in AFt, and 1170 cm^−1^ and 3675 cm^−1^ are assigned to the absorption peaks of AFm. The characteristic absorption peak of [SO_4_] belongs to AFt, which is assigned to AFm, and that for gypsum is at 1120 cm^−1^. In addition, the absorption peaks of [C=O] may belong to thaumasite and calcite partly. The measured wave number of the functional group differs from the theoretical data, which may be due to the crystal form and impurity ion. Figure 10 also shows that the admixture and erosion form can affect the wave number of erosion products, which result in the migration of the wave number and the intensity of absorption peaks. The existence of AFm may be related to the concentration of sulfate solution. The deeper the erosion depth, the lower the concentration of sulfate solution in the pores of the specimen. Therefore, the sulfate ion in the pores reacts with CAH and produces AFm. In fact, XRD spectra can be used to examine the crystal and minicrystal, but FTIR can be used to characterize amorphous products. The above results reveal that the main erosion products are AFt, AFm, gypsum, and some double salts, and erosion products, including AFt, AFm, and thaumasite, can coexist together. Because of the similar crystal structure of AFt and thaumasite, the corresponding diffraction peaks almost overlap [19,43,44]. It is therefore difficult to ensure the existence of them. However, FTIR analysis can be applied as an effective method for distinguishing AFt and thaumasite [45]. Therefore, the combination of XRD and FTIR can avoid the shortcomings of a single test method.

### 3.6. ESEM-EDS Analysis of Microstructure and Morphology of Specimen

(1) Effects of admixture and solution concentration on microstructure of specimen

**Figure 11:**
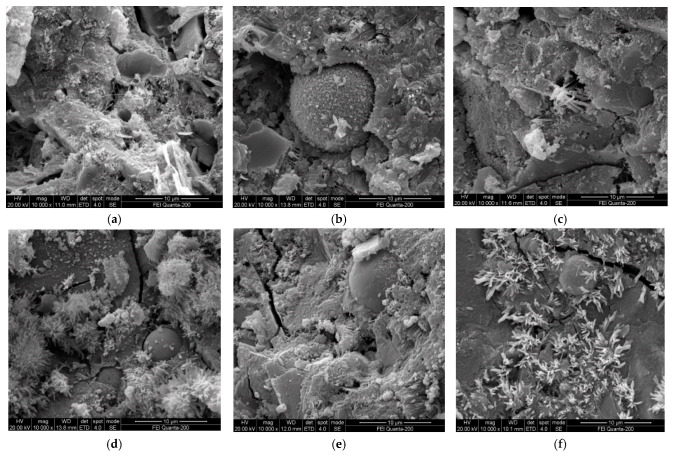

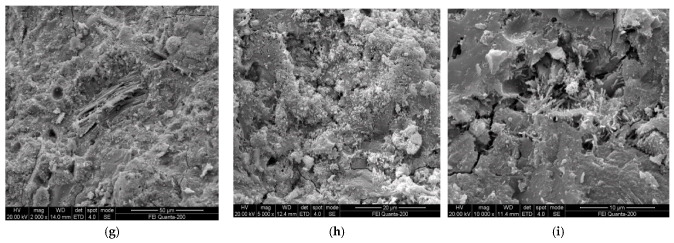
ESEM analysis of microstructure and morphology of specimen. (**a**) KB, (**b**) F5, (**c**) S5, (**d**) C1-F5, (**e**) C30-F5, (**f**) C30-F10, (**g**) C1-S5, (**h**) C30-S5, (**i**) C30-S10.

The influences of admixture and the concentration of solution on the microstructure and morphology of the specimen were researched, and the corresponding ESEM spectra of specimens subjected to sulfate attacks for 4 months are shown in Figure 11.

The effects of the kind and content of admixture on the properties of specimens are manifested as effects on the microstructure, pore characteristics, category, and morphology of hydration products, as shown in Figure 11a–c. The main hydration products of the specimen without admixture (KB) are hexagonal plate CH, rodlike AFt, gelatinous CSH, and CAH. Plenty of CH undergoes directed cleavage and is enriched in the pores, as shown in Figure 11a. The CH amount and porosity of specimens containing slag and fly ash are lower than those of the control sample (KB). The sphere surface of fly ash and particles of slag are eroded by CH and generate flocculent products, as shown in Figure 11b,c. This is because the active components of admixture are activated and reacted with the hydration products of cement. Plenty of CH that has undergone direct cleavage is expended and eliminated, and the microstructure and composition of the specimen are improved. Therefore, the appropriate amount of the admixture substitution of cement can optimize the properties of the specimen. The microstructure becomes more compact and the amount of the directed cleavage of CH decreases with an increasing concentration of sulfate solution, and many needlelike and rodlike AFt products are generated, as shown in Figure 11a,b,d,e. Moreover, Figure 11f shows that many bulky AFt products are observed from the specimen with a large amount of fly ash. Compared with that containing fly ash, the specimen containing slag has a denser microstructure and less porosity, as shown in Figure 11a,g,h,i. The microstructure of the specimen containing slag becomes denser with an increasing concentration of sulfate solution. Micro-cracks and bulky AFt can be observed when the specimen was attacked by a sulfate solution with a high concentration, as shown in Figure 11e,f,h,i. This may be caused by the expansive substances, i.e., AFt and gypsum, produced by sulfate attacks, which results in damage and cracks. The higher the concentration of sulfate solution, the greater the number of erosion products. The variations in the microstructure and erosion products of the sulfate erosion specimen manifest as a change in macroscopic strength.

(2) Effects of erosion forms on microstructure and morphology of specimen

**Figure 12:**
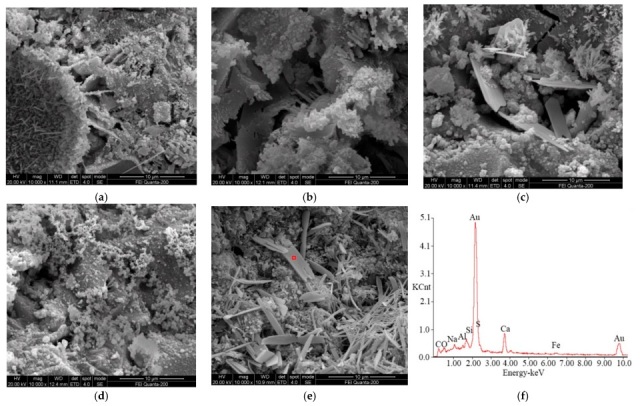
ESEM-EDS analysis of specimen subjected to sulfate attack in semi-immersion form. (**a**) B30-F5, (**b**) B10-F5, (**c**) B30-S5, (**d**) B10-S5, (**e**) B30-F5, (**f**) EDS analysis of B30-F5.

The effects of the complete and semi-immersion forms on the microstructure, morphology, and kind of sulfate erosion products were researched. Figure 12 shows the ESEM-EDS spectra of specimens attacked by 10% sulfate solution for 4 months in the semi-immersion form.

Plenty of needlelike, rodlike, and platelike substances with clear edges and smooth surfaces are generated when the specimens containing admixture are subjected to sulfate attacks in the semi-immersion form. The length and diameter of rodlike crystals are about 3 μm~5 μm and 0.5 μm, respectively. Correspondingly, the length, width, and thickness of platelike substances are 10 μm, 5 μm, and 1 μm, respectively. In Figure 12e,f, the ESEM-EDS analysis of specimens subjected to sulfate attacks indicates that the main elements of rodlike substances are Ca, Al, O, and S. Combining the morphology features, AFt can be observed. Compared with the specimen attacked by 10% sulfate solution, the erosion products of the specimen attacked by saturated sulfate solution have a bulky dimension and high crystallinity, as shown in Figure 12a,c. The above results reveal that the sulfate solution concentration has a notable influence on the microstructure, composition, and kind of erosion products. The higher the concentration of sulfate solution, the stronger the evaporation and condensed effect occurring on the surface of the specimen. Therefore, more and larger expansive substances are generated in the pores of the specimen. Compared with the complete immersion form, i.e., Figure 11e, the microstructure of the specimen containing fly ash attacked by sulfate in the semi-immersion form is loosened, as shown in Figure 11a. The erosion products of the specimen containing slag are platelike and granular, and the dimension of the rodlike substance is small. The difference in the kind and morphology of the erosion products of specimens containing admixture may be caused by differences in the compositions of slag and fly ash, which manifest as differences in the mechanical properties and morphology of salt crystallization, as shown in Figure 6 and Figure 7. The EDS analysis shown in Figure 12f is a point-by-point elemental analysis conducted on the typical erosion products in Figure 12e. KCnt in Figure 12f represents the intensity of the signal, which is initially employed to identify the elements present and their relative quantities. From the analysis of the elements in Figure 12f, it can be seen that the sheet-like products in Figure 12e are mainly composed of elements such as Ca, S, Al, and O and are preliminarily identified as erosion products such as calcium sulfate. As discussed above, a conclusion can be drawn that the concentration of sulfate solution, erosion form, and admixture can have significant influences on the kind and morphology of erosion products.

## 4. Conclusions

(1) The effects of solution concentration, the kind and substitute amount of admixture, erosion form, and age on the strength of sulfate erosion specimens were studied, and the corresponding relationship between the strength and influencing factors of the specimens was proposed. The results indicate that the compressive and flexural strengths of the specimens subjected to sulfate attacks for 4 months decrease with an increasing substitute amount of admixture, and the corresponding strength values are lower than those of the control sample. There is a GaussMod function between the substitute amount of fly ash and the flexural strength of the specimens attacked by sulfate, and a Boltzmann function can be used to characterize the variation between the substitute amount of slag and the compressive strength of the specimen.

(2) The strength of specimens containing fly ash attacked by saturated sulfate solution increases first and then decreases with an increasing substitute amount of fly ash. In the semi-immersion erosion form, the strength of specimens containing admixture subjected to sulfate attack is lower than that of the control sample. Compared with fly ash, slag has a more significant influence on the strength of specimens. The differences in the kind, form, and morphology of the erosion products of specimens subjected to sulfate attacks are related to the composition elements and active components of admixture. Admixture can observably change the microstructure and morphology of hydrates. The morphologies of salt crystallization on the surface of specimens with fly ash are fibrous and flocculent, but they are nubbly and granular for specimens containing slag.

(3) The variations in the phase composition of specimens before and after sulfate attacks mainly manifest as the appearance and disappearance of diffraction peaks and variations in peak intensity and width. The main erosion products of specimens attacked by sulfate are AFt, CAH, CSH, and gypsum. The XRD spectra indicate that the intensity of the diffraction peak of AFt is enhanced, but it is weakened for CH. The composition of materials can affect the kind of erosion products, and some compound salts including glauberite and sodium gypsum are observed. The FTIR and XRD analyses indicate that AFt, AFm, and thaumasite can coexist together.

(4) ESEM-EDS analysis indicates that sulfate attack and admixture can affect the microstructure, porosity, and morphology of hydration products. When slag and fly ash are added to specimens, the amount of CH and number of cracks decrease, and the corresponding microstructure becomes denser. Plenty of rodlike and platelike erosion products of specimens containing admixture are generated in the semi-immersion form.

## Figures and Tables

**Table 1:** Physical properties of cementitious materials.

Items	Average Diameter/µm	Specific Surface Area/(m^2^/kg)	Bulk Density /(g/cm^3^)	Initial Setting Time/min	Final Setting Time/min	Water Requirement for Standard Consistency/%
PC	34.6	345.2	1.35	172	251	28
Fly ash	41.3	332.7	0.78	-	-	-
Slag	31.7	432.5	1.18	-	-	-

## Data Availability

The original contributions presented in this study are included in the article. Further inquiries can be directed to the corresponding author.

## Appendix A

**Table A1:** Symbols and abbreviations.

Symbols and Abbreviations	Meaning	Symbols and Abbreviations	Meaning
*x*	Admixture substitute amount for cement	S5	Specimen with slag substitute amount of 5% for cement
*y*	Strength of the specimen attacked by sulfate	F30	Specimen with fly ash substitute amount of 30% for cement
*a*, *b*, *c*, *d*, *A*, *B*, *D*, *x*_0_, *W*, *E*, *t*_0_, *y*_0_, *w*, *x*_*c*_	Fitted parameters	C1-F5	Specimen containing 5% fly ash attacked by 1% sulfate solution in complete immersion form
CH	Calcium hydroxide	C30-F5	Specimen containing 5% fly ash attacked by saturated sulfate solution in complete immersion form
PC	Portland cement	C30-F10	Specimen containing 10% fly ash attacked by saturated sulfate solution in complete immersion form
AFt	Ettringite	C1-S5	Specimen containing 5% slag attacked by 1% sulfate solution in complete immersion form
CSH	Calcium silicate hydrate	C30-S5	Specimen containing 5% slag attacked by saturated sulfate solution in complete immersion form
CAH	Calcium aluminate hydrate	C30-S10	Specimen containing 10% slag attacked by saturated sulfate solution in complete immersion form
KB	Control sample	C30-KB	Control sample attacked by saturated sulfate solution in complete immersion form
XRD	X-ray diffraction	B10-F5	Specimen containing 5% fly ash attacked by 10% sulfate solution in semi-immersion form
FTIR	Fourier transform infrared spectroscopy	B30-S5	Specimen containing 5% slag attacked by saturated sulfate solution in semi-immersion form
ESEM	Environment scanning electron microscope	B10-S5	Specimen containing 5% slag attacked by 10% sulfate solution in semi-immersion form
F5	Specimen with fly ash substitute amount of 5% for cement	B30-F5	Specimen containing 5% fly ash attacked by saturated sulfate solution in semi-immersion form
